# Engineered mucoperiosteal scaffold for cleft palate regeneration towards the non-immunogenic transplantation

**DOI:** 10.1038/s41598-021-93951-w

**Published:** 2021-07-16

**Authors:** M. I. Rizzo, L. Tomao, S. Tedesco, M. Cajozzo, M. Esposito, C. De Stefanis, A. M. Ferranti, D. Mezzogori, A. Palmieri, G. Pozzato, M. Algeri, F. Locatelli, L. Leone, M. Zama

**Affiliations:** 1 Plastic and Maxillofacial Surgery Department, Bambino Gesù Children’s Hospital, IRCCS, Rome, Italy; 2 Department of Pediatric Onco-Hematology and Cell and Gene Therapy, Bambino Gesù Children’s Hospital, IRCCS, Rome, Italy; 3 Telea Biotech e Telea Electronic Engineering, Sandrigo, VI Italy; 4 Research Laboratories, Histology Core Facility, Bambino Gesù Children’s Hospital, IRCCS, Rome, Italy; 5 Department of Neuroscience, Università Cattolica del Sacro Cuore, 00168 Rome, Italy; 6 Department of Cardiovascular, Endocrine-Metabolic Diseases and Aging, National Institute of Health, Rome, Italy; 7 Department of Gynecology/Obstetrics & Pediatrics, Sapienza University of Rome, Rome, Italy; 8 Fondazione Policlinico Universitario A. Gemelli IRCCS, 00168 Rome, Italy

**Keywords:** Stem cells, Medical research, Engineering

## Abstract

Cleft lip and palate (CL/P) is the most prevalent craniofacial birth defect in humans. None of the surgical procedures currently used for CL/P repair lead to definitive correction of hard palate bone interruption. Advances in tissue engineering and regenerative medicine aim to develop new strategies to restore palatal bone interruption by using tissue or organ-decellularized bioscaffolds seeded with host cells. Aim of this study was to set up a new natural scaffold deriving from a decellularized porcine mucoperiosteum, engineered by an innovative micro-perforation procedure based on Quantum Molecular Resonance (QMR) and then subjected to in vitro recellularization with human bone marrow-derived mesenchymal stem cells (hBM-MSCs). Our results demonstrated the efficiency of decellularization treatment gaining a natural, non-immunogenic scaffold with preserved collagen microenvironment that displays a favorable support to hMSC engraftment, spreading and differentiation. Ultrastructural analysis showed that the micro-perforation procedure preserved the collagen mesh, increasing the osteoinductive potential for mesenchymal precursor cells. In conclusion, we developed a novel tissue engineering protocol to obtain a non-immunogenic mucoperiosteal scaffold suitable for allogenic transplantation and CL/P repair. The innovative micro-perforation procedure improving hMSC osteogenic differentiation potentially impacts for enhanced palatal bone regeneration leading to future clinical applications in humans.

## Introduction

Cleft lip and palate (CL/P) is the most common congenital craniofacial anomaly resulting from a disturbed embryonic development^1^. It is a complex malformation that affects both soft and hard tissues, extending from upper lip to gingiva, anterior and posterior palate. With a reported prevalence rate of approximately 1 in 700, it may lead to a wide spectrum of problems, such as limitations in chewing and swallowing, which impair normal feeding skills, development of abnormal speech mechanisms, maxillary growth and dental disorders that inevitably bring to lack of self-esteem in these little patients^1–3^.

Different and controversial are the current protocols existing for the surgical correction of CL/P, which vary regarding optimal timing and surgical procedures. Specifically, a critical issue in cleft surgery concerns, more than the mucocutaneus cleft, the repair of the hard palate's bone interruption. Regardless of the technique used, the multilayer, tension-free closure of the hard palate remains the less satisfying result of the surgical correction when considering long-term complications.

Tissue engineering can bring to a solution in facing the structural complexity of the craniofacial district providing biocompatible, non-toxic and non-immunogenic scaffolds to use as templates for tissue regeneration or organ transplantation^4^. In the last years, specifically, tissue or organ-decellularized biomaterials are emerging as the best option to generate scaffolds that mimic missing body parts. Decellularization permits the elimination of the primary tissue cellular contents leaving the native extracellular matrix (ECM) intact and allows then a second phase of recellularization where scaffolds are seeded with host cells to create a non-immunogenic scaffold, and a transplantable tissue repopulated with recipient cells in order to reduce the possibility of rejection^5–7^.

For this purpose, different cell types, combined with various biomaterials, have been used^8–11^. Among them, mesenchymal stromal cells (MSCs) emerged as a particularly useful biological source thanks to their ability to differentiate in vitro into osteocytes, adipocytes and chondrocytes and to modulate the immune response through the secretion of molecules and extracellular vesicles with anti-inflammatory and proangiogenic properties^12–16^.

With the present study we explore the possibility to produce engineered porcine mucoperiosteum scaffolds to be tested for their in vitro properties. In particular, porcine palates were decellularized, either treated or not with a perforation protocol by using Quantum Molecular Resonance (QMR), and recellularized with human bone marrow-derived MSCs (hBM-MSCs) in two different culture conditions. The obtained data set the basis for the next animal experimentation, in order to bring the protocol, in the near future, towards the human setting.

## Results

### Palate decellularization and poration

**Figure 1:**
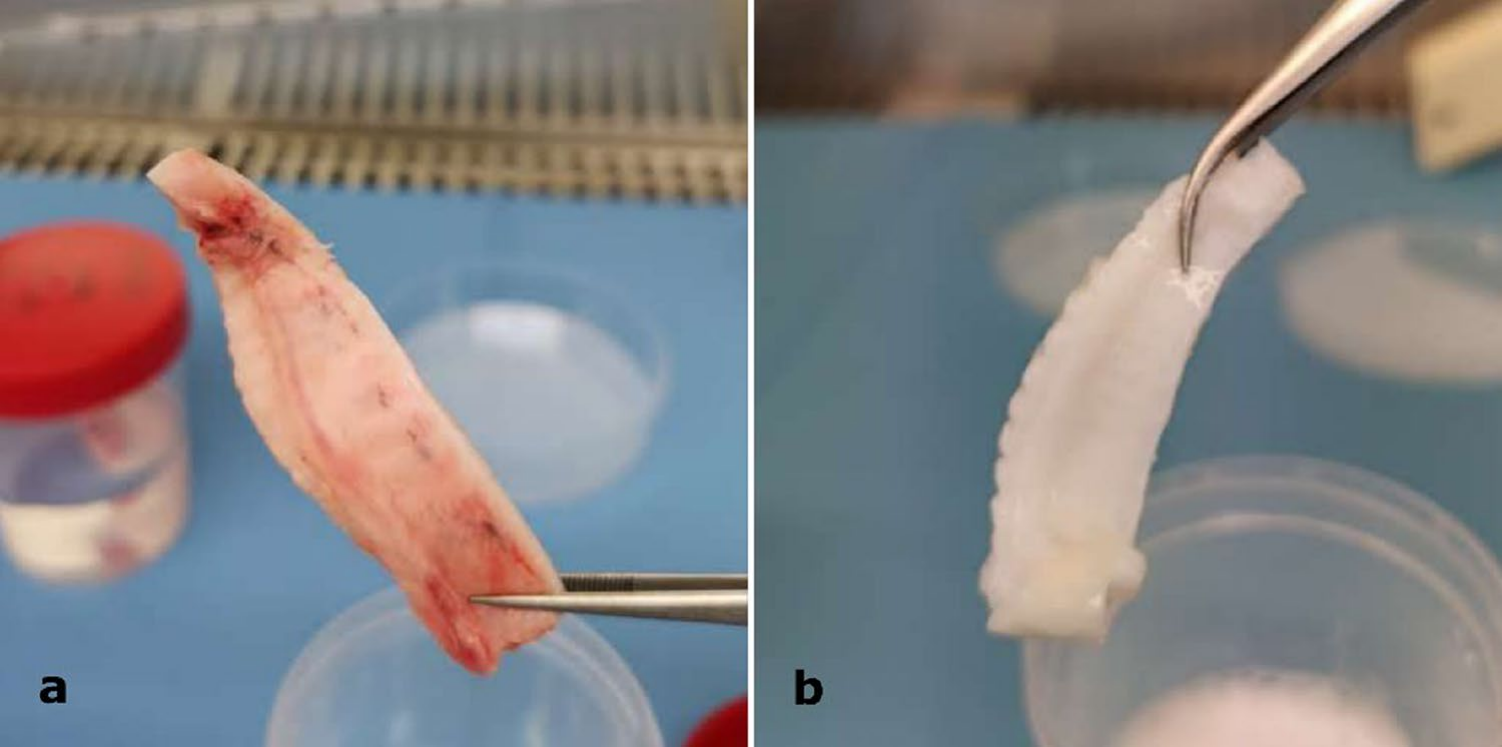
(**a**,**b**) Representative images of (**a**) non-decellularized and (**b**) decellularized hemi-palates. Each palate was washed in ultrapure water, treated in 4% sodium deoxycholate and then with DNase-I to be finally rinsed in ultrapure water. In order to remove decellularization reagents, samples were then washed with increasing percentages of denatured ethanol and finally rehydrated in ultrapure water. The macroscopic examination revealed that the color of the decellularized samples turned to white, while the non-decellularized ones showed a typical light pink shade.

**Figure 2:**
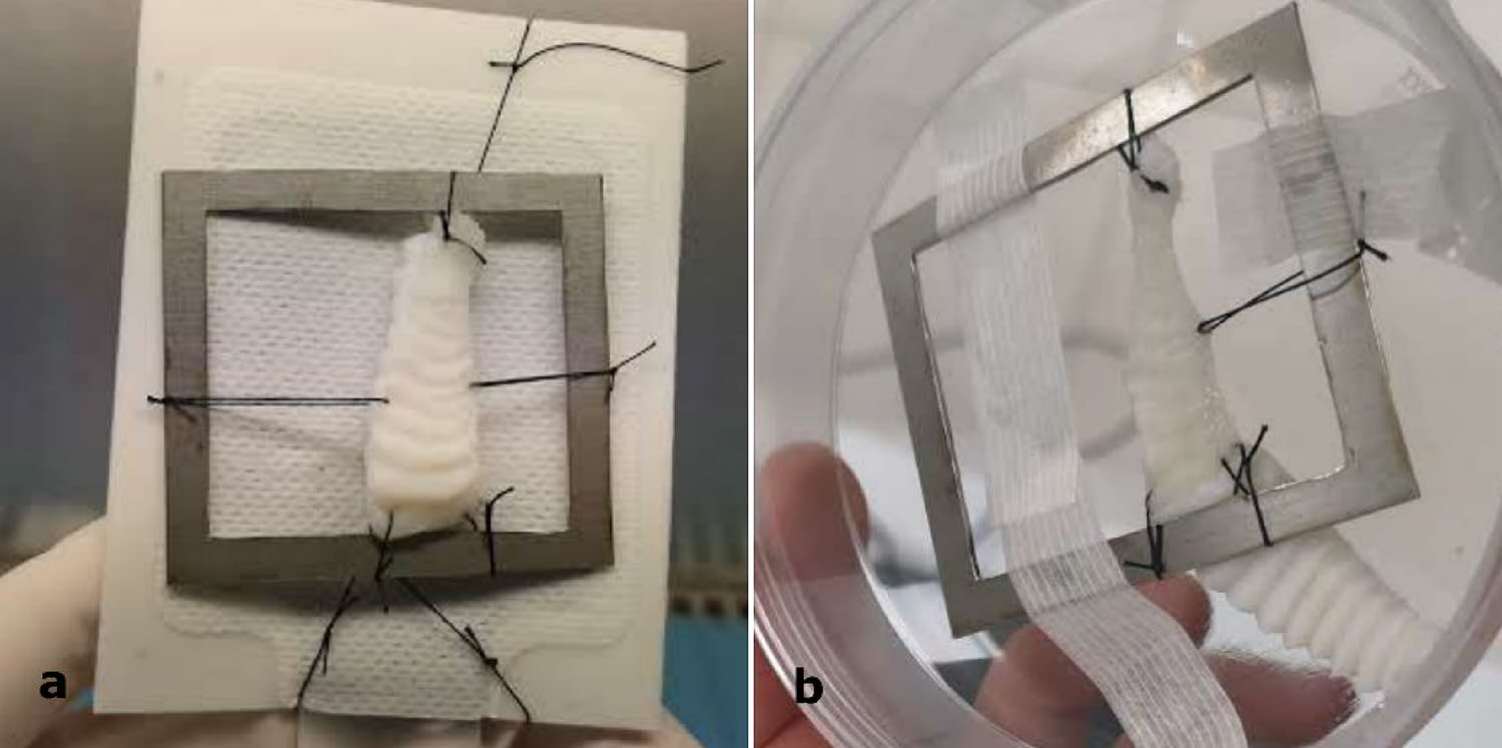
(**a**,**b**) Representative images of (**a**) non-pored and (**b**) pored scaffolds. Half of the decellularized scaffolds was treated with a microscopic patented perforative treatment using a Quantum Molecular Resonance current generator (VESALIUS) connected to a 300 μm-diameter needle, generating up to 1000 micropores/cm^2^. The macroscopic examination showed a slightly increased stiffness and a decreased thickness in the pored samples, which also showed a more transparent appearance compared to the non-pored ones.

Palates decellularization was analyzed through both macroscopic examination and DNA quantification. The first observation after decellularization treatment was the color of the palates, with decellularized samples that turned to white instead of a light pink typical of the non-decellularized ones, as expected from a complete acellular matrix (Fig. 1). Differences were noted also between pored and non-pored scaffolds, with a slightly increased stiffness and a decreased thickness in the pored samples, which also showed a more transparent appearance (Fig. 2).

DNA quantification revealed minimal traces of the donor’s DNA for all the decellularized scaffolds analyzed. All the specimens analyzed showed values ranging from a minimum of 17.91 to a maximum of 38.89 ng of dsDNA per 1 mg of fresh tissue, settling below the critical value of 50 ng/mg (Table 1). Average DNA residual calculated from all the decellularized samples was 28.39 ng/mg, compared to the value of 303.43 ng/mg obtained from the fresh specimens, with a percentage residual content of 9.36%. DNA quantification also exhibited minimal differences in DNA residuals among the specimens belonging to P and NP group (respectively 28.77 and 28.01 ng/mg).

**Table 1:** DNA quantification in fresh and decellularized palates.

Palate	DNA residuals (ng/mg)		
	Fresh	Decellularized NP	Decellularized P
1	653.06	17.91	28.97
2	210.00	37.43	34.29
3	159.62	19.41	38.89
4	307.78	36.00	30.30
5	199.92	32.12	11.65
6	290.19	25.21	28.52
*p-*value		< 0.05	< 0.05
		NS	

Table 1 showed the DNA quantification to reveal donor’s DNA residuals in fresh and decellularized (non-pored, NP and pored, P) scaffolds. All the decellularized specimens showed DNA levels below the critical value of 50 ng/mg. In both groups of decellularized specimens a significant reduction of DNA content, respect to fresh specimens, was observed (p < 0.05, student’s T-test). However, this reduction was minimal comparing the NP and the P groups.

Based on established criteria for effective decellularization^17^, it is common to find donor DNA residuals (as long as it is below the critical value of 50 ng/mg) and other remnants, such as nuclei content, cytoplasmic proteins, extracellular components or detergents used. In fact, decellularization techniques cannot completely remove cellular material, however, it is possible and necessary to perform a quantitative analysis of residual cellular components. The advantage of this decellularization technology consists in preserving vascular integrity, 3D architecture and mechanical properties of the scaffold, all well represented in our matrices, which also show a very low value of donor DNA residuals.

**Figure 3:**
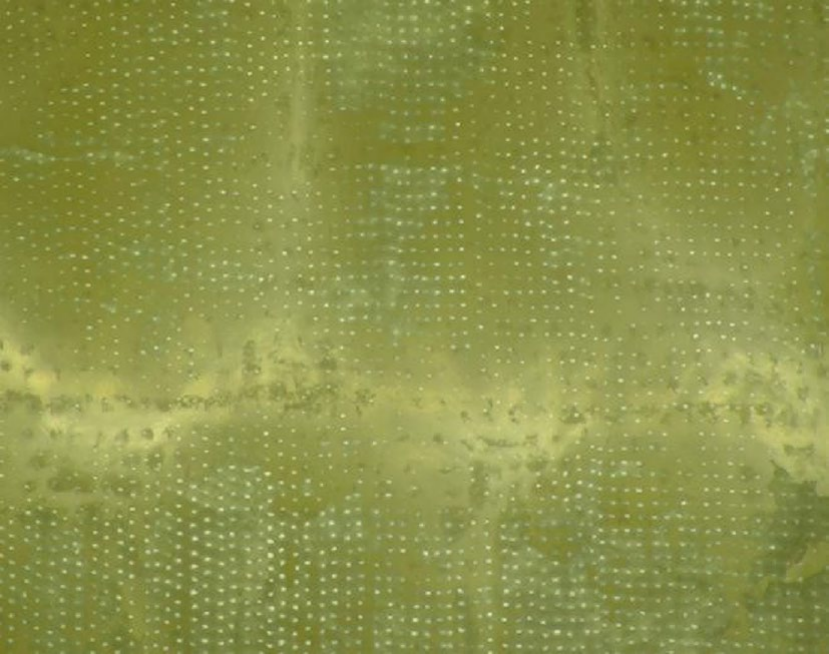
Low magnification optical microscope image to evaluate the efficiency of poration in a P sample. The presence of pores in the entire surface was confirmed by the greater passage of light through them.

The efficacy of palate poration made on samples belonging to the P group was confirmed by microscopical examination. At different magnifications, the presence of pores in the entire surface was clearly evidenced by the greater passage of light in correspondence of them (Fig. 3).

### MSC isolation and characterization

**Figure 4:**
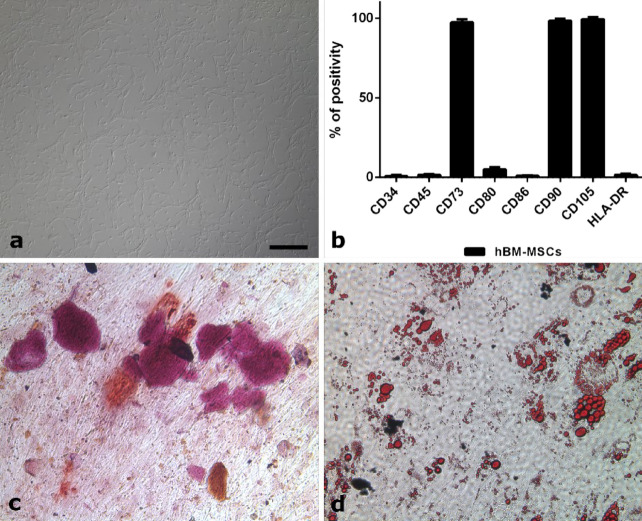
(**a**–**d**) MSCs characterization after in vitro expansion. (**a**) Cells, isolated from the bone marrow of a healthy donor, display the typical spindle-shaped morphology of MSCs. (**b**) Immunophenotyping by flow cytometry reveals expression of MSCs markers (CD73, CD90, and CD105) and lack of hematopoietic markers (CD34, CD45, CD80, CD86, and HLA-DR). (**c**) After incubation with the appropriate medium, the isolated cells were able to differentiate into osteoblasts as revealed by Alizarin Red staining (Sigma-Aldrich), which highlights calcium depositions. (**d**) At the same time, the isolated cells were also able to differentiate into adipocytes, as detected by Oil Red O (Sigma-Aldrich), which makes fat droplets visible. All the figures in the panel are the same scale; bar—200 µm.

MSCs were successfully isolated from a healthy donor BM samples. The ex vivo expanded cells displayed the characteristic spindle-shaped morphology of MSCs (Fig. 4a) and the cytometric analysis showed that they expressed the typical MSCs markers (CD73, CD90 and CD105), lacking the hematopoietic markers (CD34, CD45, CD80, CD86 and HLA-DR) (Fig. 4b). Moreover, all the isolated cells were able to differentiate into both osteoblasts (Fig. 4c) and adipocytes (Fig. 4d).

### Microscopic evaluation of the seeded scaffolds

**Figure 5:**
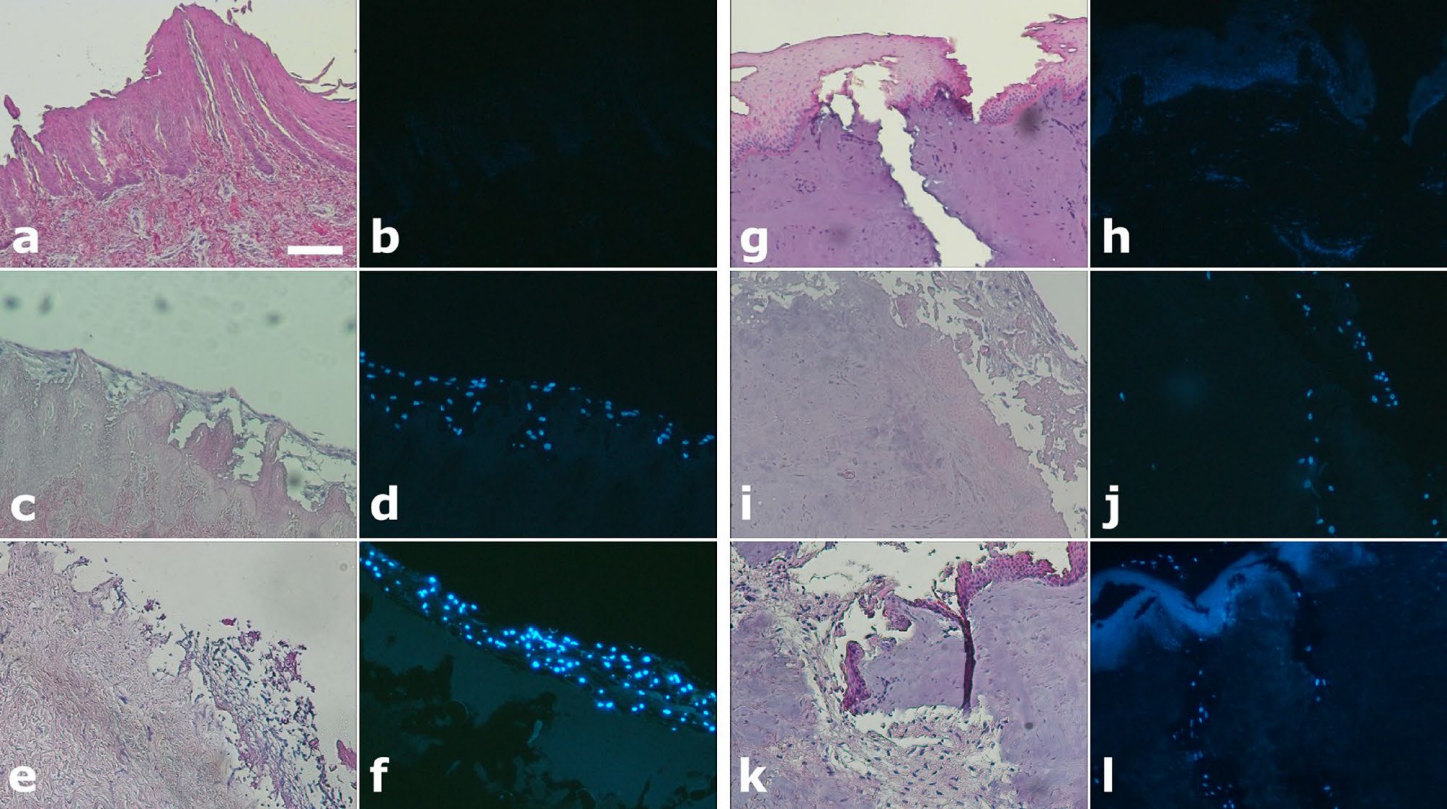
(**a**–**l**) Light and fluorescence microscopy images of non-pored (**a**–**f**) and pored (**g**–**l**) scaffolds stained either with HE (**a**,**c**,**e**,**g,i**,**k**) or DAPI (**b**,**d**,**f**,**h**,**j**,**l**), before and after seeding with MSCs cultured with FBS. (**a**,**b**,**g**,**h**) Pre-seeding images of both non-pored (**a**,**b**) and pored (**g**,**h**) scaffolds reveal no presence of MSCs. (**c**,**d**,**i**,**j**) After 7 days post-seeding, MSCs have adhered superficially to the non-pored scaffold (**c**,**d**) while in the pored one (**i**,**j**) a repopulation of the channels was already observed. (**e**,**f**,**j**,**k**) After 14 days post-seeding, MSCs were even more present on the surface of the non-pored scaffold (**e**,**f**), while they were able to penetrate deeper and with a great extent in the pored one (**k**,**l**). All the figures in the panel are the same scale; bar—100 µm.

Pored and non-pored decellularized scaffolds were incubated with MSC for 14 consecutive days. The light/fluorescence microscopy images taken pre- and post-seeding (d7 and d14) confirmed the presence of residual porcine nuclear material in the scaffold, although at a small extent, as also revealed by the DNA quantification analysis. At the same time no MSCs were observed in the pre-seeding scaffolds, while the images clearly showed that the seeded MSCs (d7 and d14) successfully adhered to the surface of the scaffold in all conditions (pored or not and using both FBS and hPL). The images also showed that MSCs were able to colonize the channels of the pored scaffolds in both FBS (Fig. 5) and hPL culture conditions (Supplemental Figure 1).

**Figure 6:**
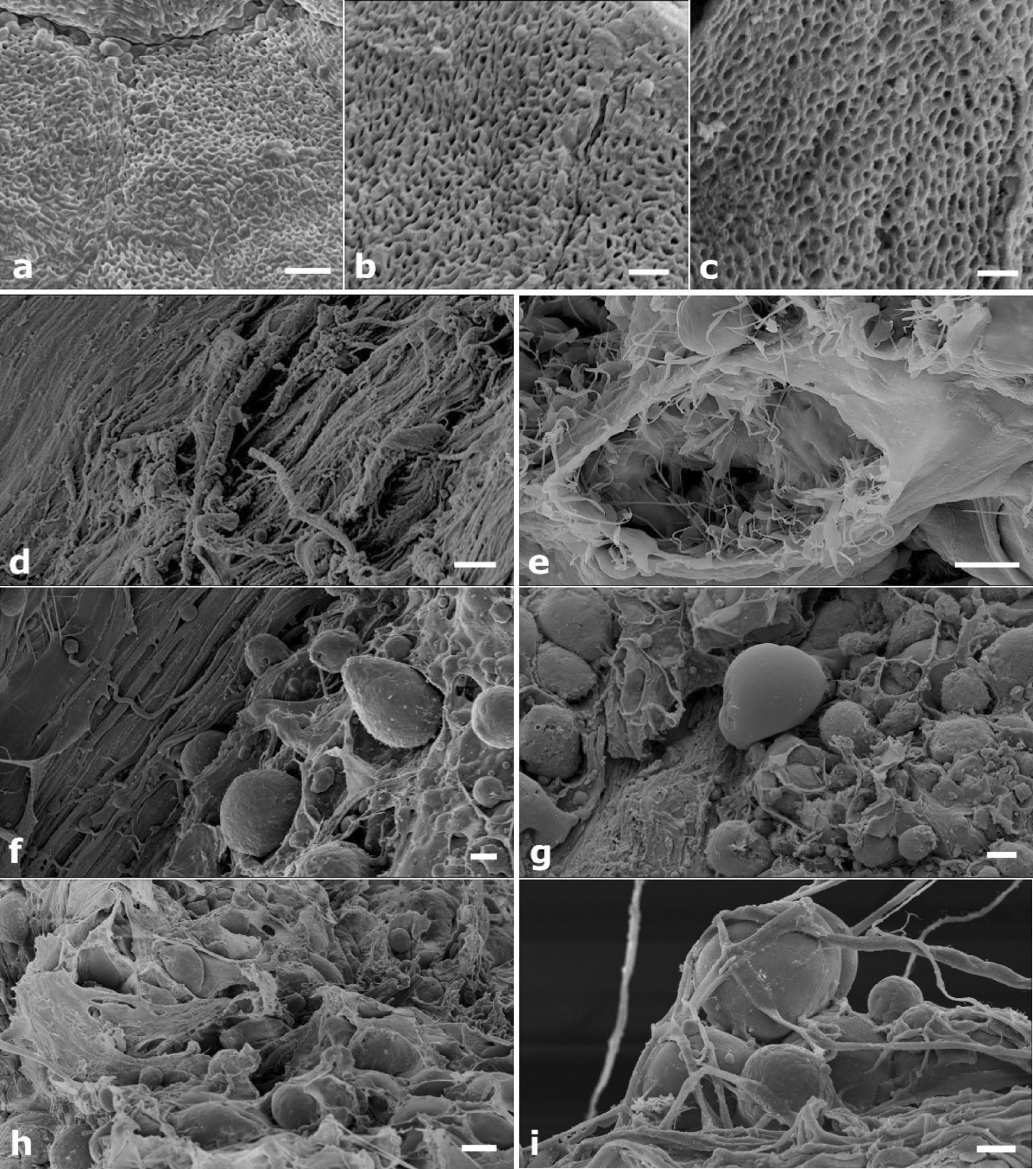
(**a**–**i**) SEM images of the bone scaffold. (**a**–**c**) The tissue texture is unaltered in the natural untreated bone sample (**a**), it appears well preserved in the decellularized sample (**b**) and shows no apparent modification or damage after microporation (**c**). (**d**,**e**) More in detail, the decellularized and micro-pored scaffold reveals a cell-free collagen matrix (**d**) and well preserved cellular niches (**e**). (**f**,**g**) 7 days after seeding, MSCs have colonized the decellularized and pored structure, with cells randomly dispersed and well anchored to micropores (**f**), however they remain superficially (**g**). (**h**,**i**) 14 days after seeding, the scaffold is covered with cells (**h**) that grow inwardly in a multilayered disposition (**i**). Bars: (**a**)—2 μm; (**b**)—1 μm; (**c**)—1 μm; (**d**)—20 μm; (**e**)—5 μm; (**f**)—10 μm; (**g**)—20 μm; (**h**)—20 μm; (**i**)—10 μm.

To better characterize the bioscaffolds and to deepen the effectiveness of the decellularization and poration protocol, SEM analysis was performed. Low magnification micrographs of the decellularized palates showed a mesh of intact collagen fibers similar to the ultrastructure of the natural untreated bone sample (Fig. 6a,b). A well-preserved tissue texture with a cell-free matrix and without any apparent modification or damage was also evident after micro-perforation (Fig. 6c). Cellular niches were recognizable throughout the perforated scaffold, providing a suitable microenvironment for cell engraftment and differentiation (Fig. 6d,e). At d7 the MSCs colonization of the decellularized and pored structures was already evident, with cells randomly disperse on the scaffold surface as well as within the micropores (Fig. 6f,g). At d14, perforated scaffolds were covered by multi-layered cells grown inwardly (Fig. 6h,i). These images were consistent with those obtained by light microscopy.

### Gene and protein expression profile of seeded scaffolds

**Figure 7:**
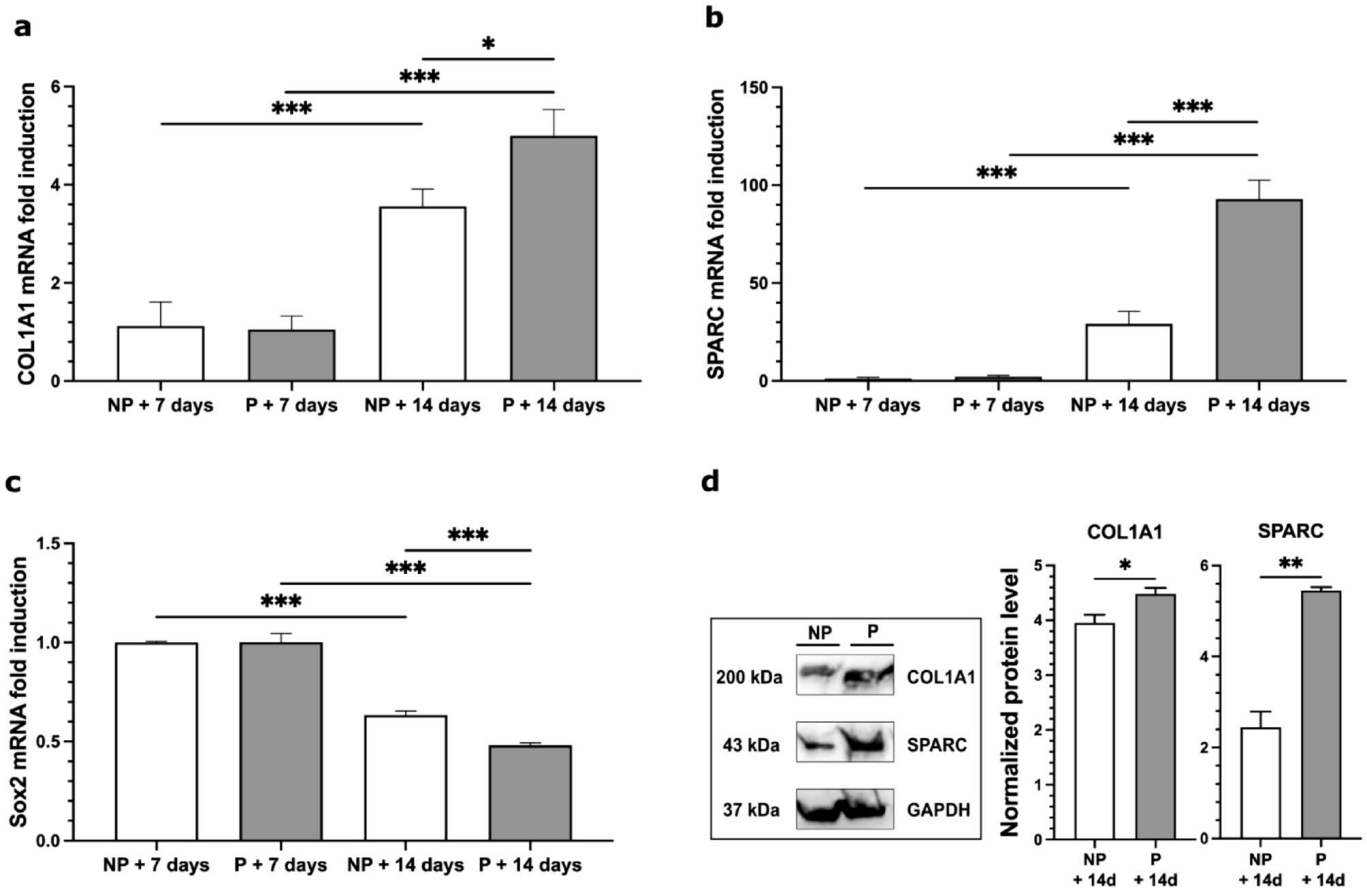
(**a**–**d**) mRNA relative expression analysis of COL1A1, SPARC and Sox2 genes and WB assay of COL1A1 and SPARC proteins. (**a**–**c**) mRNA relative expression analysis (mean + SEM) of COL1A1, SPARC and Sox2. (**a**,**b**) mRNA relative expression analysis of the osteogenic differentiation genes COL1A1 (i.e. pro-alpha1 chains of type I collagen) and SPARC (i.e. secreted protein acidic and rich in cysteine) in non-pored (NP) and pored (P) scaffolds, 7 and 14 days after seeding. Expression levels of both genes are increased in the d14 scaffolds in comparison to the d7 ones. (**c**) Conversely, a progressive decrease of Sox2 mRNA level in non-pored (NP) and pored (P) scaffolds was detected at d7 and d14, confirming the differentiation of MSCs during this time window. Real-time PCR analysis was performed in triplicate (n = 6 pig per experimental group; statistics by two-way ANOVA and Bonferroni post hoc). Data are expressed as mean ± SEM. *p < 0.05; ***p < 0.001. (**d**) Immunoblot analysis and bar graphs showing the levels of COL1A1 and SPARC in non-pored (NP) and pored (P) scaffolds seeded with hBM-MSCs for 14 days. Cropped images are from samples run on the same gel. Pored scaffolds showed a significant increase in both COL1A1 and SPARC protein levels compared to the non-pored samples (*n* = 3; statistics using unpaired Student’s *t* test). GAPDH was used as loading control. Data are expressed as mean ± SEM. *p < 0.05; **p < 0.01. Positive and negative controls, as well as molecular size markers and full-length blots are presented in Supplementary Figure 2.

To further assess the positive effect of the micro-perforative treatment on MSC seeding of decellularized scaffolds, we performed mRNA relative expression analysis of the osteogenic differentiation genes COL1A1 (i.e. pro-alpha1 chains of type I collagen), SPARC (i.e. secreted protein acidic and rich in cysteine) BGLAP (i.e. bone gamma-carboxyglutamic acid-containing protein) and the stemness gene Sox-2 (i.e. SRY (sex determining region Y)-box 2). In line with the above results a significant enhancement of COL1A1 and SPARC gene expression was detected in the pored scaffolds compared to the non-pored samples. Moreover, both COL1A1 and SPARC mRNA levels were increased in the d14 scaffolds in comparison with the d7 ones (*F*_*3.28*_ = 33.328 for COL1A1*,* comparison for time factor: d14 vs. d7 *p* < 0.001, comparison for treatment within day14: P *vs.* NP *p* = 0.017; *F*_*3.28*_ = 39.900 for SPARC, comparison for time factor: d14 *vs*. d7 *p* < 0.001; comparison for treatment within d14: P *vs.* NP *p* < 0.001; Fig. 7a,b). Conversely, a progressive decrease of Sox2 mRNA level was detected at d7 and d14, confirming a progressive differentiation of MSCs during this time window (*F*_*3.28*_ = 370,887 for Sox2*,* comparison for time factor: d14 *vs.* d7 *p* < 0.001; comparison for treatment: P *vs.* NP *p* = 0.004; comparison for treatment within d14: P *vs.* NP *p* < 0.001; Fig. 7c). No significant differences between FBS and hPL MSC differentiation protocols were observed in mRNA levels of the selected genes at any time points (data not shown).

Protein expression of both COL1A1 and SPARC was also evaluated by WB analysis on tissue extracts of the same samples used for mRNA quantification. Accordingly, 14 days after MSC seeding, pored scaffolds showed a significant increase in both COL1A1 and SPARC protein levels compared to the non-pored samples (P vs. NP *p* = 0.048 and 0.001 respectively; Fig. 7d).

## Discussion

CL/P is a congenital craniofacial malformation, which often requires multiple surgeries throughout the child’s growth^18–20^. Different surgical procedures are currently used for CP repair, depending on the type and width of the cleft. All of them aim to recreate a multilayered closure of the mucosal surfaces with reposition of the abnormal velar musculature, without any correction of the bone cleft^18,21^. However, none of these procedures can be considered definitive and often patients require further corrective surgeries. With an incidence ranging from 0 to 35%, occurrence of palatal fistula is one of the commonest complications that can be related to a mechanism of repair under tension and lack of available tissue due to the wide clefts^22–25^. Furthermore, as reported by several studies, surgical procedures, causing palatal wound contraction, can impair maxillary bone growth and bring to abnormal dental arch relationships^26–28^.

Given these conditions, tissue-engineered materials (natural polymers, synthetic polymers, tissue- organ-biomaterials) can serve as sources of scaffolds to be used in conjunction with regenerative medicine for craniofacial defects reconstruction^29^. In the last decades, polymeric scaffolds have been used as a support for conventional surgery, to repair craniomaxillofacial defects^30,31^. However, the majority of the materials tested has often brought to unsatisfactory results and so researchers are trying to develop new strategies to produce less inert scaffolds, showing better interaction and deeper integration with the host. Finally, further advances have been made, through the use of regenerative medicine, by seeding scaffolds with selected cell populations that could enhance scaffold engraftment. This approach seems to achieve good results both in animal models and in human settings^31–35^.

In order to find new methodological approaches in this complex scenario, we developed a mimetic natural scaffold based on a decellularized porcine mucoperiosteum, engineered by perforative treatment. This decellularized bioscaffold is able to preserve the structural, biochemical, and biomechanical properties of the native tissue, therefore providing a suitable environment for subsequent hMSCs repopulation by osteogenic differentiation.

The decellularization process deprived the extracellular matrix (ECM) of the native cells and genetic material to produce a natural, non-immunogenic scaffold. Even a minimal trace of donor DNA in the scaffold could elicit the host immune response and lead to tissue rejection or fibrous encapsulation^36^. Therefore, researchers have stated a limit value of 50 ng dsDNA per mg ECM weight as the fundamental criterion for assessing the effectiveness in the removal of DNA remnants^37^. In this work, we evaluated the success of the mucoperiosteal decellularization by both macroscopic examination and DNA quantification. All the decellularized specimens appeared white, which is typical of tissues presenting a loss of cellular components. Molecular analysis revealed the presence of only small amounts of donor DNA, which never exceeded the limit value of 50 ng of dsDNA per mg of ECM tissue, neither in NP nor in P groups. These data confirmed the efficacy of decellularization protocol for palate-derived tissues, although the achieved results could be further implemented by applying further changes in the decellularization protocol (*e.g.* performing additional cycles of DNase-I treatment). The minimal differences in DNA residual content between P and NP samples demonstrated that the micro-perforative treatment with QMR did not affect the efficacy of decellularization, even if increased stiffness in specimens belonging to the P group was observed. Nevertheless, after micro-perforation no sign of necrosis or thermal damage was observed in P samples, suggesting that the ECM could be positively accepted by the host for a future surgery.

As described above, both P and NP palate scaffolds were then subjected to the recellularization protocol, which consisted in seeding the decellularized scaffolds with MSCs previously isolated from the BM of a healthy donor. The advantages of using these cells depend on their ability to orchestrate inflammatory processes which guide all the phases of tissue regeneration, their high resistance to neoplastic transformation after in vitro exposure to physical and chemical stress, and their capacity to improve the tolerance towards the non-self^38–41^. Moreover, the differentiation properties of BM-MSCs could be important in sustaining the osteogenic and chondrogenic composition of the tissues to be restored^42^. The hBM-MSCs used in the present study were successfully isolated and ex vivo expanded for several passages. Before seeding, these cells were characterized and tested for their ability to differentiate into adipocytes and osteocytes. The recellularization of palate scaffolds with hBM-MSCs was performed by using FBS or hPL as medium supplement, and the obtained results were evaluated at day 7 and day 14. Light and fluorescence microscopy showed the presence of porcine cell traces in all the decellularized scaffolds, confirming the DNA analysis and suggesting to add one DNase-I cycle to our decellularization protocol. The BM-MSCs seeded on both NP and P scaffolds exhibited good biocompatibility but their distribution was not homogeneous: MSCs seeded on NP scaffolds had adhered only superficially, whereas those seeded on P palates appeared to sprout and penetrate deeper into the tissue. However, since the micro-perforative treatment could not be equally displayed in all the histological sections, the microscopic analysis showed that some areas were richer in cells than others, suggesting that tissue re-population could depend on poration extent. Lastly, microscopic analysis revealed that both medium supplements used for cell culturing and seeding (i.e. FBS and hPL) allowed the growth and adhesion of MSCs in the ex vivo expansion and seeding phases. This aspect is of particular relevance considering that the use of xeno-free products is essential for the future application of these engineered scaffolds in the human setting.

Our SEM analysis confirmed clinical effectiveness of the micro-perforative treatment by allowing in vitro MSC engraftment throughout chemotaxis stimulation, cell adhesion and osteoblast proliferation and differentiation. It has already been established that decellularization methods depend on tissue-specific factors, such as cell number, matrix micro-architecture and surface ligand sites^43^. These features include ECM fiber orientation and network topology that are not always well preserved during this process. In this respect, our decellularized and pored bioscaffold showed the presence of bioactive ECM mesh, which provided a favorable microenvironment for proper MSC engraftment, as revealed by ultrastructural analysis. Moreover, this mucoperiosteum engineered by perforative treatment revealed increased osteoinductive potential for mesenchymal precursor cells. The hBM-MSCs seeded on pored specimens exhibited a higher degree of osteogenic differentiation compared to non-pored groups. Our molecular results showed a significant increase in the expression levels of both COL1A1, and SPARC, especially on day 14. These osteoblast-related genes are considered master regulators of osteoblast differentiation and early transcription factors for the determination of osteoblast lineage^44,45^. Specifically, SPARC overexpression inhibited cell proliferation inducing hBM-MSC osteoblast differentiation^46^. We also observed a reduction in the expression of the stemness gene Sox2, indicating the capability of the proposed scaffold to induce cell differentiation also in the absence of any other microenvironmental signal. Comparable molecular data were also obtained by Mattioli-Belmone et al.’s research on a demineralized bone matrix seeded human umbilical cord-derived MSCs^47^.

Based on the above results, we can hypothesize that in vivo transplantation of porcine mucoperiosteum engineered by QMR perforative treatment and seeded with hBM-MSCs may act as a recruiter of mesenchymal precursors as well as a differentiation factor, inducing higher expression of osteogenic markers and promoting palate repair.

## Concluding remarks and future perspectives

Advances in tissue or organ biomaterials represent a great potential for the creation of engineered tissues with comparable composition, structure and mechanical functionality to native hard palate. With this study, we have explored in vitro properties of engineered porcine mucoperiosteum scaffolds. We have developed a new protocol to efficiently decellularize and repopulate this bioscaffold with hBM-MSCs. These cells, together with the low level of donor DNA and the natural structure of ECM, will facilitate both mucous and bone regeneration, maintenance as well as reduction of immunogenicity following transplantation. The experience gathered with the perforative treatment, based on QMR, has led to positive results, showing a higher cell adhesion and a three-dimensional distribution of MSCs within the scaffold, these two features could prove effective in tissue regeneration in vivo.

This study lays the foundations for a new frontier in CP repair. Engineered mucoperiosteum scaffolds, repopulated with recipient MSCs, could not only naturally integrate in the cleft palate, but also solve the issues related to life-long immunosuppressive therapy which affect patients receiving allogenic tissues.

Our next research steps are aimed at reproducing in vivo the results obtained in vitro. In this perspective, our experimentation will be focused on in vivo transplantation of engineered mucoperiosteum scaffolds into recipient pigs to effectively restore the integrity of hard palate without risk of rejection. For this purpose, palates will be initially harvested from corpses of donor pigs and subjected to the protocols of decellularization described above. Sampling of bone marrow from recipient pigs will be collected to isolate selectively the BM-MSCs which will be used for the recellularization phase. A court of recipient pigs, previously subjected to surgical procedure in order to induce iatrogenic palate cleft, will serve as recipient animals for engineered mucoperiosteum transplantation. One month after implantation, the non-immunogenic mucoperiosteum engraftment and bone regeneration will be evaluated. Long-term studies will be carried out to verify the implant-tissue interaction and hierarchical structure of regenerated bone in order to finally turn them into a clinically viable strategy.

The outcome will be significant both from a scientific point of view for the progress in the field of regenerative medicine and tissue engineering and from a socio-medical point of view, representing the chance to offer an innovative treatment for malformative, post-oncological and post-traumatic pathologies.

## Materials and methods

### Ethics

The study was conducted in accordance with ethical standards and principles expressed in the Declaration of Helsinki. Informed consent was obtained from all donors included in the study and approved by the local Ethics Committee of the Bambino Gesù Children’s Hospital.

All animal procedures were fully compliant with Italian (Ministry of Health guidelines, Legislative Decree No. 116/1992) and European Union (Directive No. 86/609/EEC) legislations on animal research.

All experimental protocols were carried out in strict accordance with the approved guidelines of Italian legislation.

### Pigs and palates harvesting

All animal procedures were fully compliant with Italian (Ministry of Health guidelines, Legislative Decree No. 116/1992) and European Union (Directive No. 86/609/EEC) legislations on animal research.

The methods were carried out in strict accordance with the approved guidelines.

The mucoperiosteum of the hard palate of six pigs (pig 1–6) obtained from a local abattoir were stored in dry ice in Styrofoam container and transported to the laboratory within 1–2 h of their collection. Randomly selected pieces from each fresh palate were collected for DNA quantification before dry storage at − 80 °C. All the palates were then subjected to the standard decellularization protocol and they were longitudinally divided into two identical hemi-palates, resulting in a total of 12 samples, randomly divided into two groups, the pored (P) and the non-pored (NP), with only those belonging to the P-group being further manipulated for the perforative treatment, as stated above.

### Palate decellularization

Each palate was washed in ultrapure water and 2% Penicillin–Streptomycin (P/S, Sigma-Aldrich) for 48 h at 4 °C in static conditions and incubated five times for 4 h at room temperature in 4% sodium deoxycholate (BioXtra ≥ 98.0%—Sigma-Aldrich). Samples were then treated with 2000 Kunitz Unit (KU) of DNase-I (Warthington) in 1 M NaCl for 3 h at and incubated at 37 °C (5×) and then rinsed in ultrapure water and 2% P/S. In order to remove decellularization reagents, samples were washed with increasing percentages of denatured ethanol (ACS Reagent, ≥ 99.8%, without additive—Honeywell) and then rehydrated overnight in ultrapure water. Scaffolds were then stored in Phosphate Buffered Saline (PBS, Sigma-Aldrich) and 1% P/S at 4 °C. Efficiency of decellularization protocol was evaluated by DNA quantification.

### Scaffold perforative treatment

After decellularization, each palate was cut longitudinally in order to divide them into two samples for a comparative analysis: one half was left intact, while the other half was subjected to a microscopic patented perforative treatment (patented by Telea Biotech) using the VESALIUS current generator, based on Quantum Molecular Resonance (QMR) technology (property of Telea Electronic Engineering Srl)^48^, connected to a 300 μm-diameter needle mounted on a 3-axis Cartesian robot (Yamaha model RCX240) handpiece. Procedures were performed in class-II biological safety cabinet under aseptic conditions. The treatment allows to make micropores on biological tissues with adjustable dimensions and densities up to 1000 pores/cm^2^ without causing burning phenomena or affecting the surrounding ECM structure, thus allowing to significantly increase the available surface for cell attachment^49^. Its efficacy it had already been demonstrated in a recent esophageal reconstruction study on piglets with the same technique. Pores’ creation and dimensions were analyzed with digital microscope (Vision Engineering model EVO Cam) at different magnifications^50^.

### DNA quantification

Double stranded DNA (dsDNA) was quantified in fresh samples and in both decellularized only and decellularized/pored scaffolds. dsDNA was isolated using the DNeasy Blood and Tissue kit (Qiagen) following manufacturer’s instructions. DNA was quantified by using a BioPhotometer Plus (Eppendorf). Optical densities at 260 nm and 280 nm were used to estimate DNA purity and yield.

### Palate recellularization

#### Cell cultures

MSCs were isolated and cultured as previously described^40^. Briefly, mononuclear cells were isolated from residual cells of a healthy donor, who donated BM for transplantation at the Bambino Gesù Children’s Hospital, and after obtaining a written informed assent/consent. Cultures were maintained at 37 °C in a humidified atmosphere, containing 5% CO_2_. After 48-h adhesion, non-adherent cells were removed and culture proceeded with culture medium being replaced twice a week. MSCs were harvested, after reaching ≥ 80% confluence and were propagated at 4 × 10^3^ cells/cm^2^.

#### Immunophenotype

MSCs were phenotypically characterized by flow cytometry using different fluorophore-conjugated monoclonal antibodies specific for CD34, CD45, CD73, CD80, CD86, CD90, CD105 and HLA-DR. (BD PharMingen, San Diego, CA). Analysis of cell populations was performed by means of direct immunofluorescence with a FACSCanto flow-cytometer (BD PharMingen) and data were calculated using the FACSDiva software (Tree Star, Inc. Ash-land, OR).

#### Differentiation capacity

The differentiation potential of MSCs was assessed as previously described^40^. Osteogenic and adipogenic differentiation were evaluated following calcium and fat droplets deposition via Alizarin Red and Oil Red O (Sigma-Aldrich, St Louis, MO) staining, respectively.

#### Seeding

Before seeding, part of each scaffold was excised as control. MSCs from passage 2 (P2) and 4 (P4) were harvested, counted and resuspended at a concentration of 1 × 10^6^/ml. Cells were then seeded on each decellularized scaffold (pored or non-pored) immobilized on the bottom of a petri dish, previously washed and re-equilibrated in medium. After 2 h enough medium to cover the seeded scaffolds was added, maintaining the culture for 14 days and performing 3 re-seeding as previously described. Part of each scaffold were excised at day 7 (d7) and at the end of culturing (d14) to be tested as described afterwards.

### Ultra structural analysis

#### Light and fluorescence microscopy

Decellularized scaffold samples, excised pre- and post-seeding (d7 and d14), were fixed in 10% formalin for 24 h and embedded in paraffin. Slices of 2 µm thickness were then cut and placed on positive charged slides and stained either with Dapi or with hematoxylin/eosin. Slides were the analyzed by light microscopy with Eclipse E600 (Nikon) the following days.

#### Scanning electron microscopy—SEM

Both pre- and post-seeding scaffolds (d7 and d14) were fixed in 1% glutaraldehyde in 0.1 M cacodylate buffer, post-fixed in 1% osmium tetroxide, dehydrated in increasing ethanol concentrations (50%, 70%, 80%, 90% and 100% for 10 min each) and hexamethyldisilazane (HDMS)-dried. The specimens were then mounted on aluminum stubs, gold-sputtered by the Agar High Resolution Coater equipment, and analyzed by SUPRA 25 SEM (Zeiss, Germany) microscope.

### Molecular analysis

#### Real-time quantitative reverse transcription PCR (qRT-PCR)

All reverse transcription-quantitative PCR (RT-qPCR) experiments were performed as previously described^51^. Quantitative RT-PCR was performed in triplicates using inventoried TaqMan Gene expression assays purchased from Applied Biosystems. Relative mRNA levels for genes of interest were normalized to Glyceraldehyde 3-phosphate dehydrogenase (GAPDH), hypoxanthine guanine phosphoribosyltransferase (HPRT), TATA-binding protein (TBP) and beta glucuronidase (GUSB) taken as housekeeping genes and calculated by using the 2^−ΔΔCt^ method. Three independent experiments were performed with the ABI 7500 Sequence Detection System Analyzer for RT-qPCR (Applied Biosystems). Results were expressed as mean fold changes induced by post-seeded treatments and comparing d7 and d14 scaffolds. ID codes for TaqMan probes against gene targets analyzed in this work are provided in Table 2.

**Table 2:** Taqman assays used for RT-qPCR.

Target gene	Taqman assay ID
BGLAP	Hs01587814_g1
COL1A1	Hs00164004_m1
GAPDH	Hs01548420_m1
GUSB	Hs00939627_m1
Hprt	Hs02800695_m1
Sox2	Hs00415716_m1
SPARC	Hs00234160_m1
Tbp	Hs00427620_m1

Table 2 showed the list of TaqMan primers and probes used for the gene expression assays (Applied Biosystems) in real-time RT-qPCR analysis.

#### Western blotting

The MSC-seeded scaffolds were lysed in ice-cold lysis buffer (NaCl 150 mM, Tris–HCl 50 mM pH 8, EDTA 2 mM) containing 1% Triton X-100, 0.1% SDS, 1× protease inhibitor cocktail (Sigma-Aldrich, St. Louis, MO, USA), 1 mM sodium orthovanadate (Sigma-Aldrich, St. Louis, MO, USA), 1 mM sodium fluoride (Sigma-Aldrich, St. Louis, MO, USA), and 1 mM phenylmethylsulfonyl fluoride (Sigma-Aldrich, St. Louis, MO, USA). The lysate was sonicated for 5 min using a Diagenode Bioruptor Standard Waterbath Sonicator (medium level). Samples were spun down at 13,000×*g* at 4 °C for 15 min. Supernatant was quantified for protein content using the Bradford method (DC Protein Assay; Bio-Rad, Hercules, CA, USA). Equal amounts of proteins were diluted in Laemmli buffer, boiled and resolved using SDS-PAGE. Primary antibodies anti-COL1A1, anti-SPARC, anti-alpha-tubulin (Cell Signaling Technology Inc., Danvers, MA, USA) and anti-glyceraldehyde 3-phosphate dehydrogenase, GAPDH (Abcam, Cambridge, UK) were incubated overnight and signals were revealed with HRP-conjugated secondary antibodies (Cell Signaling Technology Inc., Danvers, MA, USA) and chemiluminescent substrates (Cyanagen, Bologna, BO, Italy). UVItec Cambridge Alliance was used to detect and quantify the luminescent signal of the protein bands. Expression levels of the target proteins were normalized to those of the housekeeping protein GAPDH (loading control) in each lane. Representative images of WB were cropped for data presentation in the main figure. Full length WB images are shown in Supplementary material.

#### Statistical analysis

Sample sizes were chosen with adequate power (0.8) according to results of pilot data sets, including our own, which used similar methods. Sample estimation and statistical analyses were performed using SigmaPlot 14 software. Data were first tested for equal variance (Brown-Forsythe) and normality (Shapiro–Wilk test) and the appropriate statistical tests were chosen. The statistical tests used (i.e., Student’s t-test, two-way ANOVA and Bonferroni t-test) are indicated in the main text and in the corresponding figure legends for each experiment. Sample size (n = 3/group) is indicated in the figure legend and represents independent experiments from different animals. Significance in normally distributed samples was analyzed using a two-tailed Student’s t test and two-ways analysis of variance (ANOVA) for post hoc pair wise comparisons including the control group. Post-hoc multiple comparisons were performed with Bonferroni correction. All statistical tests were two-tailed and the level of significance was set at 0.05. Results are shown as mean ± SEM.

## Supplementary Information

Supplementary Information.
