# Supplementary material for: Engineered mucoperiosteal scaffold for cleft palate regeneration towards the non-immunogenic transplantation
Source: Sci Rep. 2021 Jul 16;11:14570. doi: 10.1038/s41598-021-93951-w (PMC8285425; doi:10.1038/s41598-021-93951-w)
Supplement: Supplementary file 1 — Supplementary Information. [file 41598_2021_93951_MOESM1_ESM.pdf]

Engineered Mucoperiosteal Scaffold for Cleft Palate Regeneration towards the Non-immunogenic Transplantation

M. I. **Rizzo**[1]* *and* L. **Tomao**[2]*, S. **Tedesco**[3], M. **Cajozzo**[1], M. **Esposito**[1], C. **De Stefanis**[4], A. M. **Ferranti**[5], D. **Mezzogori**[5], A. **Palmieri**[6], G. **Pozzato**[3], M. **Algeri**[2], F. **Locatelli**[2,7]* *and* L. **Leone**[5,8]* *and* M. **Zama**[1]*

Affiliations

[1] Plastic and Maxillofacial Surgery Department, Bambino Gesù Children's Hospital, *IRCCS*, Rome, Italy.
[2] Department of Pediatric Onco-Hematology and Cell and Gene Therapy, Bambino Gesù Children's Hospital, *IRCCS*, Rome, Italy.
[3] Telea Biotech e Telea Electronic Engineering, Sandrigo (VI), Italy.
[4] Research Laboratories, histology core facility, Bambino Gesù Children 's Hospital, IRCCS, Rome, Italy
[5] Department of Neuroscience, Università Cattolica del Sacro Cuore, 00168 Rome, Italy
[6] Department of Cardiovascular, Endocrine-metabolic Diseases and Aging, National Institute of Health, Rome, Italy
[7] Department of Gynecology/Obstetrics & Pediatrics, Sapienza University of Rome, Rome, Italy.
[8] Fondazione Policlinico Universitario A. Gemelli IRCCS, 00168 Rome, Italy
* These Authors contributed equally.

Corresponding author

Dr. Lucia Leone
e-mail: lucia.leone@unicatt.it
Department of Neuroscience, Università Cattolica del Sacro Cuore, 00168 Rome, Italy
Fondazione Policlinico Universitario A. Gemelli IRCCS, 00168 Rome, Italy

Supplemental Figure 1

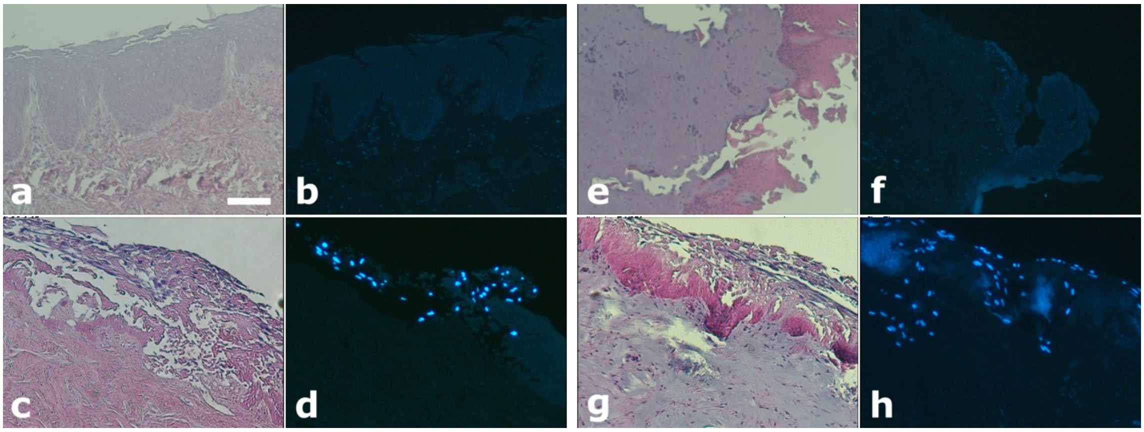

Supplemental Figure 1 (**a** – **h**) Light and fluorescence microscopy images of non-pored (**a** – **d**) and pored (**e** – **h**) scaffolds stained either with HE (**a**, **c**, **e**, **g**) or DAPI (**b**, **d**, **f**, **h**), before and after seeding with MSCs cultured with hPL. (**a**, **b**, **e**, **f**) Pre-seeding images of both non-pored (**a** – **b**) and pored (**e** – **f**) scaffolds reveal no presence of MSCs. After 14 days post-seeding, MSCs have superficially repopulated the non-pored scaffold (**c – d**) while in the pored one (**g – h**) a repopulation of the channels, with a good extent, was observed. All the figures in the panel are the same scale; bar – 100 μm.

Supplemental Figure 2

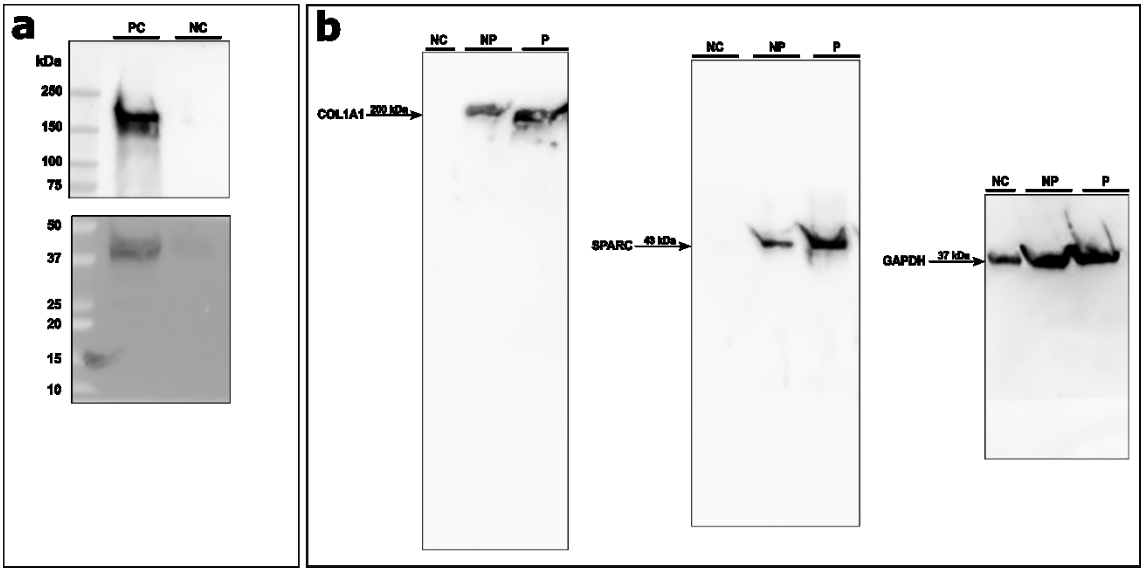

Supplemental Figure 2 (a – b) Representative Western blot (WB) images of COL1A1 and SPARC in positive and negative controls, and in non-pored (NP) and pored (P) samples. (**a**) WB of positive control (PC) and negative control (NC) samples immunoblotted with anti-COL1A1 and anti-SPARC to evaluate the specificity of the antibodies e their predicted molecular weight (**b**) Full length WB of NC, NP and P samples seeded with hBM-MSCs for 14 days and immunoblotted for COL1A1 and SPARC, and for GAPDH as loading control.
